# Supplementary material for: Tailoring neuromuscular dynamics: A modeling framework for realistic sEMG simulation
Source: PLoS One. 2025 Jun 12;20(6):e0319162. doi: 10.1371/journal.pone.0319162 (PMC12161536; doi:10.1371/journal.pone.0319162)
Supplement: Attachment — Submitted filename: Response To Plos Com Bio Editor.pdf [file pone.0319162.s001.pdf]

PLOS Computational Biology Editor Comments:

Thank you very much for submitting your manuscript "Tailoring Neuromuscular Dynamics: A Modeling Framework for Realistic sEMG Simulation" (PCOMPBIOL-D-24-01033) for consideration at PLOS Computational Biology. As with all papers, your manuscript was reviewed by members of the editorial board. Based on our initial assessment, we regret that we will not be pursuing this manuscript for publication at PLOS Computational Biology.

While the work was thorough, there were some substantial concerns. Most importantly, the focus on individual muscle fibers as the control element of skeletal muscle (rather than the motor unit), is without physiological support. Also, as this work was proposed as a realistic framework for simulating surface EMG, the failure to include the characteristics of the surface electrodes themselves (e.g., their surface areas) is a critical oversight. Lastly, the model was not used to address any questions.

Authors' Response:

We appreciate the feedback provided by the PLOS Computational Biology Editor. However, we would like to clarify some aspects of our work that may address the concerns raised.

First Comment: “The focus on individual muscle fibers as the control element of skeletal muscle (rather than the motor unit) is without physiological support.”

Our work does not propose individual muscle fibers as the control element of skeletal muscle. Instead, our model establishes the muscle fiber as the most elemental functional unit for muscle contraction. Each muscle fiber is defined as an independent programmatic object. Additionally, we model the motor neurons as the elements that group muscle fibers into motor units and the motor control system responsible for recruiting motor units and determining their firing rates. This aligns well with our current understanding of the neuromuscular control system. Defining each muscle fiber programmatically allows us to define their physical properties independently, offering higher configurability of the muscular system (providing a better approach to account for inter-subject variabilities). This creates a framework where single-fiber phenomena like muscle fatigue and the decay of individual fibers during aging can also be configured and studied. Models that use the motor unit as the minimal unit of activation lack this level of configurability. We believe our model represents an advancement in muscle activity modeling.

This information is clearly stated in the introduction and emphasized throughout the manuscript.

<u>Second Comment:</u> “Also, as this work was proposed as a realistic framework for simulating surface EMG, the failure to include the characteristics of the surface electrodes themselves (e.g., their surface areas) is a critical oversight.”

In our manuscript, we compare real sEMG signals with those simulated by our model. The similarities between simulated and real signals for each participant were higher than the similarities of real signals across participants for the same tasks. This includes comparisons of both spectral and temporal features. The frequency-by-frequency analysis in Figure 5 clearly shows that the similarity between real and simulated data is higher than any other reported in the literature so far. We acknowledge that including more detailed modeling of electrodes could further enhance realism, but we do not view the current level of detail as a critical oversight. Other research published in PLOS Computational Biology, such as Pereira et al., "Anatomically accurate model of EMG during index finger flexion and abduction derived from diffusion tensor imaging," PLoS Computational Biology 15.8 (2019): e1007267, also does not include these specific features.

Our model comprises five independent elements whose functions are interconnected to simulate sEMG signals, allowing individual modifications to each element without affecting the rest. This ensures that the realism of the model can be further developed in the future. Given the current state of the art, we consider our electrode definition sufficient for achieving a high level of realism in the simulated signals (as proved by the results of our paper).

<u>Third Comment:</u> “Lastly, the model was not used to address any questions.”

As stated in the introduction of our work, the main goal of the paper was to introduce a framework for the simulation of realistic sEMG signals to the scientific community. We believe the results provided in the paper demonstrate the potential of our model to achieve this. We also explained the reason behind the development of this model in both the introduction and discussion sections. We aim to have a simulation framework that includes all elements reported to modify the frequency of sEMG signals, allowing the effects of each element to be isolated and studied. This has been an important debate within the scientific community over the last decade.

Also, our work reports significant changes in the frequency domain of simulated sEMG signals when we alter the distribution of fast and slow-twitch muscle fibers within the simulated muscle.

This was an initial result to introduce a future work we plan to undertake using this model as reference.

The paper includes a detailed explanation of each of the five elements defined in our model, their functionalities and interconnections, and how each element was parametrized before sEMG simulation. Experiments were conducted to record real sEMG and force profiles used to validate the realism of the simulated signals. This alone brought our manuscript close to 30 pages of definition and validation.

Including additional experiments to address extra scientific questions would dilute the original goal of the paper. We believe that such models should be properly presented and validated before they are used to solve specific problems, and that is what we aimed to do in this paper.

In Summary:

Although we appreciate the feedback provided by the editor at PLOS Computational Biology, we do not fully understand how the contents of our manuscript led to the comments received. We hope that the PLOS ONE review process can offer extra insights about our work.
